# Phase I trial of single-photon emission computed tomography–guided liver-directed radiotherapy for patients with low functional liver volume

**DOI:** 10.1093/jncics/pkae037

**Published:** 2024-05-10

**Authors:** Enoch Chang, Franklin C L Wong, Beth A Chasen, William D Erwin, Prajnan Das, Emma B Holliday, Albert C Koong, Ethan B Ludmir, Bruce D Minsky, Sonal S Noticewala, Grace L Smith, Cullen M Taniguchi, Maria J Rodriguez, Sam Beddar, Rachael M Martin-Paulpeter, Joshua S Niedzielski, Gabriel O Sawakuchi, Emil Schueler, Luis A Perles, Lianchun Xiao, Janio Szklaruk, Peter C Park, Arvind N Dasari, Ahmed O Kaseb, Bryan K Kee, Sunyoung S Lee, Michael J Overman, Jason A Willis, Robert A Wolff, Ching-Wei D Tzeng, Jean-Nicolas Vauthey, Eugene J Koay

**Affiliations:** Radiation Oncology, The University of Texas MD Anderson Cancer Center, Houston, TX, USA; Nuclear Medicine, The University of Texas MD Anderson Cancer Center, Houston, TX, USA; Nuclear Medicine, The University of Texas MD Anderson Cancer Center, Houston, TX, USA; Imaging Physics, The University of Texas MD Anderson Cancer Center, Houston, TX, USA; Radiation Oncology, The University of Texas MD Anderson Cancer Center, Houston, TX, USA; Radiation Oncology, The University of Texas MD Anderson Cancer Center, Houston, TX, USA; Radiation Oncology, The University of Texas MD Anderson Cancer Center, Houston, TX, USA; Radiation Oncology, The University of Texas MD Anderson Cancer Center, Houston, TX, USA; Radiation Oncology, The University of Texas MD Anderson Cancer Center, Houston, TX, USA; Radiation Oncology, The University of Texas MD Anderson Cancer Center, Houston, TX, USA; Radiation Oncology, The University of Texas MD Anderson Cancer Center, Houston, TX, USA; Radiation Oncology, The University of Texas MD Anderson Cancer Center, Houston, TX, USA; Radiation Oncology, The University of Texas MD Anderson Cancer Center, Houston, TX, USA; Radiation Physics, The University of Texas MD Anderson Cancer Center, Houston, TX, USA; Radiation Physics, The University of Texas MD Anderson Cancer Center, Houston, TX, USA; Radiation Physics, The University of Texas MD Anderson Cancer Center, Houston, TX, USA; Radiation Physics, The University of Texas MD Anderson Cancer Center, Houston, TX, USA; Radiation Physics, The University of Texas MD Anderson Cancer Center, Houston, TX, USA; Radiation Physics, The University of Texas MD Anderson Cancer Center, Houston, TX, USA; Biostatistics, The University of Texas MD Anderson Cancer Center, Houston, TX, USA; Diagnostic Imaging, The University of Texas MD Anderson Cancer Center, Houston, TX, USA; Radiology Physics, University of California, Davis, Davis, CA, USA; Medical Oncology, The University of Texas MD Anderson Cancer Center, Houston, TX, USA; Medical Oncology, The University of Texas MD Anderson Cancer Center, Houston, TX, USA; Medical Oncology, The University of Texas MD Anderson Cancer Center, Houston, TX, USA; Medical Oncology, The University of Texas MD Anderson Cancer Center, Houston, TX, USA; Medical Oncology, The University of Texas MD Anderson Cancer Center, Houston, TX, USA; Medical Oncology, The University of Texas MD Anderson Cancer Center, Houston, TX, USA; Medical Oncology, The University of Texas MD Anderson Cancer Center, Houston, TX, USA; Surgical Oncology, The University of Texas MD Anderson Cancer Center, Houston, TX, USA; Surgical Oncology, The University of Texas MD Anderson Cancer Center, Houston, TX, USA; Radiation Oncology, The University of Texas MD Anderson Cancer Center, Houston, TX, USA

## Abstract

**Background:**

Traditional constraints specify that 700 cc of liver should be spared a hepatotoxic dose when delivering liver-directed radiotherapy to reduce the risk of inducing liver failure. We investigated the role of single-photon emission computed tomography (SPECT) to identify and preferentially avoid functional liver during liver-directed radiation treatment planning in patients with preserved liver function but limited functional liver volume after receiving prior hepatotoxic chemotherapy or surgical resection.

**Methods:**

This phase I trial with a 3 + 3 design evaluated the safety of liver-directed radiotherapy using escalating functional liver radiation dose constraints in patients with liver metastases. Dose-limiting toxicities were assessed 6-8 weeks and 6 months after completing radiotherapy.

**Results:**

All 12 patients had colorectal liver metastases and received prior hepatotoxic chemotherapy; 8 patients underwent prior liver resection. Median computed tomography anatomical nontumor liver volume was 1584 cc (range = 764-2699 cc). Median SPECT functional liver volume was 1117 cc (range = 570-1928 cc). Median nontarget computed tomography and SPECT liver volumes below the volumetric dose constraint were 997 cc (range = 544-1576 cc) and 684 cc (range = 429-1244 cc), respectively. The prescription dose was 67.5-75 Gy in 15 fractions or 75-100 Gy in 25 fractions. No dose-limiting toxicities were observed during follow-up. One-year in-field control was 57%. One-year overall survival was 73%.

**Conclusion:**

Liver-directed radiotherapy can be safely delivered to high doses when incorporating functional SPECT into the radiation treatment planning process, which may enable sparing of lower volumes of liver than traditionally accepted in patients with preserved liver function.

**Trial registration:**

NCT02626312.

When treating liver tumors with high-dose ablative radiotherapy, we traditionally spare at least 700 cc of noncirrhotic nontumor liver from a hepatotoxic dose per the experience of prior studies with the aim of reducing the risk of radiation-induced liver disease (1). However, this anatomic liver volume is usually estimated with computed tomography (CT) alone without accounting for underlying liver function. This is an important distinction when treating patients who have received prior hepatotoxic chemotherapy or undergone liver resection, as some liver volume that is uninvolved by tumor may not have normal function (2-5).

Technetium-99m sulfur colloid single-photon emission CT (SPECT) is a US Food and Drug Administration–approved diagnostic tracer that images the reticuloendothelial (Kupffer) cells of the liver and has been shown to correlate with chronic liver disease severity and function (6-8). Several studies have shown it may be possible to use SPECT to identify and preferentially avoid functional liver volume during radiation treatment planning (9-11). Additionally, functional liver metrics derived from SPECT have shown promise for clinical outcome prediction and dose-response modeling (12-14).

Kirichenko et al. (15) investigated the role of SPECT in radiation treatment planning for patients with hepatocellular carcinoma and Child–Pugh B or C cirrhosis who were treated with liver ablative radiotherapy. No patients developed radiation induced liver disease or accelerated Child–Pugh class migration.

The aim of this study was to evaluate the safety of liver-directed ablative radiotherapy for patients with preserved liver function but low functional liver volume after prior hepatotoxic chemotherapy or surgical resection.

We hypothesized that incorporating functional liver SPECT into the radiation treatment planning process would allow safe delivery of ablative radiotherapy in patients with limited liver volume.

## Methods

A phase 1 trial with a 3 + 3 design was conducted to evaluate the safety of comprehensive ablative radiotherapy to liver disease using more aggressive functional nontarget liver radiation dose constraints with each level. Eligibility criteria included 1) a diagnosis of hepatocellular carcinoma, intrahepatic cholangiocarcinoma (iCCA), or liver metastasis (LM); 2) prior treatment with irinotecan or oxaliplatin chemotherapy or liver resection; and 3) a minimum functional liver volume of 400 cc as estimated by SPECT using a threshold of 40% maximum intensity (16,17). Patients with cirrhosis, prior liver-directed radiotherapy, or prior Yttrium-90 therapy were excluded. Patient demographics including age and self-reported sex were recorded.

### Preplanning and simulation procedures

**Figure 1.**
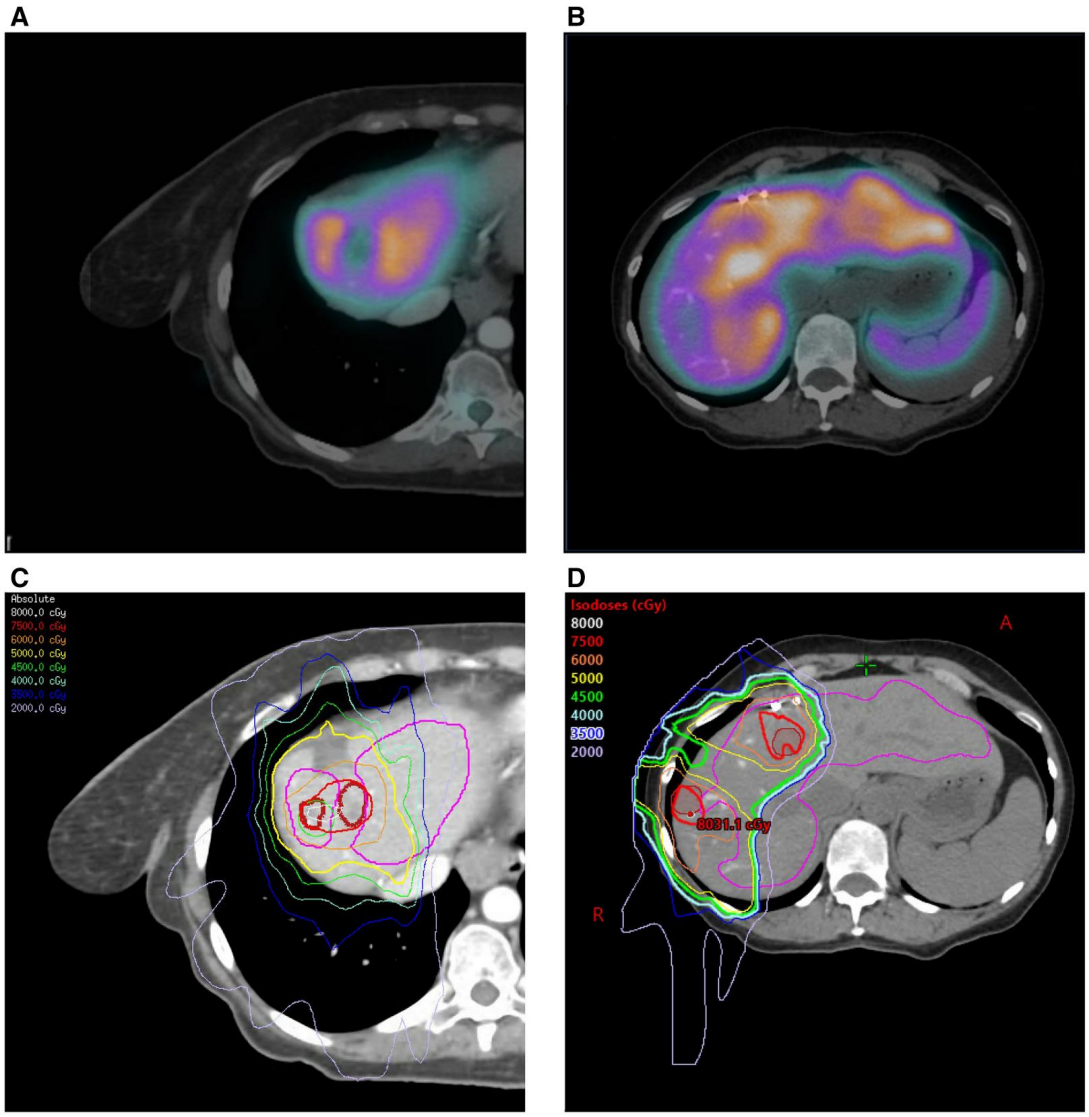
Functional liver contour per single-photon emission computed tomography (SPECT) (**A-B**) registered to the treatment planning computed tomography (CT) for incorporation into the treatment planning workflow (**C-D**). Liver volume spared below threshold dose constraint: 544 cc anatomic volume per CT and 466 cc functional volume per SPECT (**C**) and 578 cc anatomic volume per CT and 429 cc functional volume per SPECT (**D**).

For treatment planning purposes, a CT scan was obtained in the treatment position. For reproducibility, a custom immobilization device was created. Deep inspiration breath hold was preferred. Patients unable to hold their breath reliably were simulated with a 4 dimensional CT (4DCT) scan and treated free breathing with an internal target volume to encompass internal movement during all phases of the respiratory cycle. A technetium-99m sulfur colloid SPECT scan was obtained for each patient in the treatment position with their custom immobilization device. A nuclear medicine physician estimated functional liver per SPECT based on a 40% maximum intensity threshold of uptake within the liver, excluding the spleen. This threshold has been established for estimating functional liver volume in prior studies (16,17). A contour of the functional liver was generated and registered to the treatment planning CT scan (see Figure 1).

### Treatment planning

The prescription dose was 67.5-75 Gy in 15 fractions or 75-100 Gy in 25 fractions. The volumetric dose constraint for the registered functional nontarget liver per SPECT receiving less than 24 Gy for 15 fractions or less than 27 Gy for 25 fractions was determined by the dose level of trial enrollment (see Table 1). Level 0 was at least 400 cc, and level +1 was at least 300 cc. A level -1 (≥500 cc) was included if needed. Standard 15 and 25 fraction dose constraints were used for other organs at risk.

**Table 1.** Dose constraint levels^a^

Dose constraint level	Dose constraint
−1	≥500 cc functional liver per SPECT receives less than threshold dose
0	≥400 cc functional liver per SPECT receives less than threshold dose
1	≥300 cc functional liver per SPECT receives less than threshold dose

a Threshold dose for 15-fraction regimen was 24 Gy. Threshold dose for 25-fraction regimen was 27 Gy. SPECT = single-photon emission computed tomography.

### Follow-up

During treatment, patients were seen weekly by the treating radiation oncologist with lab work including blood counts, albumin, aspartate transaminase, alanine transaminase, alkaline phosphatase, blood urea nitrogen, creatinine, calcium, phosphorus, total bilirubin, total protein, electrolytes, and prothrombin time and international normalized ratio to monitor for dose-limiting toxicities, which were graded according to the Common Terminology Criteria for Adverse Events version 4.0. Patients were subsequently evaluated 6-8 weeks after completion of radiotherapy with a follow-up visit, physical exam, lab work, and imaging with abdominal CT or magnetic resonance imaging. Patients were seen every 3-4 months for follow-up for 2 years. The MD Anderson Symptom Inventory for Gastrointestinal Cancer was completed at the baseline consultation visit, weekly during treatment, and at each follow-up visit. The following dose-limiting toxicities were assessed 6-8 weeks and 6 months after completing radiotherapy: grade 3 hypoalbuminemia (<2 g/dL), increase in prothrombin time and international normalized ratio (>2.5 x upper limit of normal or >2.5 x baseline if on anticoagulation), increase in bilirubin (>3.0 to 10.0 x upper limit of normal), ascites, or grade 4 hepatic failure or any radiation-related toxicity.

### Study design

This was a prospective phase I study with a standard (3 + 3) design under a protocol approved by our institutional review board (2015-0052). The trial was registered at ClinicalTrials.gov under identifier NCT02626312. All patients signed informed consent. The primary objective of this trial was to determine the maximum dose constraints for the volume of functional liver in patients who have preserved liver function but low functional liver volume after receiving hepatotoxic chemotherapy or undergoing prior liver resection. Maximum dose constraint was defined as the highest dose constraint level at which no more than 1 patient experienced dose-limiting toxicities in 6 patients treated at that dose constraint. The first 3 patients were treated at dose level 0 (see Table 1) per either a 15-fraction regimen or 25-fraction regimen based on the ability to meet the dose constraints for the liver and other organs. If 0 of these 3 patients experienced dose-limiting toxicities, treatment was escalated to dose level +1 (see Table 1). If 1 or more of the patients in the group experienced dose-limiting toxicities, dose escalation would be stopped, and patients would be treated at the next lower dose level until 6 were treated at that dose constraint.

### Statistical analysis

Descriptive statistics were computed for variables of interest. The Kaplan–Meier method was used to estimate 1-year in-field control rate and 1-year overall survival from the date of radiotherapy completion to date of progression, death, or last follow-up. Analyses were done in Python (version 3.10).

## Results

### Patient characteristics

A total of 12 patients enrolled between February 2016 and June 2022 (Table 2). The median age was 52 years (range = 34-74 years). Seven (58%) patients were male, and 5 (42%) patients were female. All 12 had liver metastases from colorectal cancer; 3 (25%) patients had a *KRAS* mutation; and 10 (83%) patients had a *TP53* mutation.

**Table 2.** Patient characteristics^a^

Patient enrollment number	Age, y	Sex	Pathology	KRAS mutation	TP53 mutation	Prior oxaliplatin or irinotecan	Prior liver resection
1	41	M	Colorectal adenocarcinoma	No	Yes	Oxaliplatin	Yes
2	73	M	Colorectal adenocarcinoma	Yes	Yes	Oxaliplatin	Yes
3	56	F	Colorectal adenocarcinoma	No	No	Oxaliplatin	No
4	73	M	Colorectal adenocarcinoma	No	Yes	Oxaliplatin Irinotecan	Yes
5	48	F	Colorectal adenocarcinoma	No	Yes	Oxaliplatin Irinotecan	Yes
6	43	M	Colorectal adenocarcinoma	No	Yes	Oxaliplatin Irinotecan	Yes
7	46	F	Colorectal adenocarcinoma	Yes	Yes	Oxaliplatin Irinotecan	No
8	34	M	Colorectal adenocarcinoma	No	No	Oxaliplatin Irinotecan	Yes
9	64	M	Colorectal adenocarcinoma	Yes	Yes	Oxaliplatin Irinotecan	Yes
10	46	M	Colorectal adenocarcinoma	No	Yes	Oxaliplatin Irinotecan	Yes
11	56	F	Colorectal adenocarcinoma	No	Yes	Oxaliplatin Irinotecan	No
12	68	F	Colorectal adenocarcinoma	No	Yes	Irinotecan	No

a F = female; M = male.

All patients received prior hepatotoxic chemotherapy with either oxaliplatin or irinotecan. Eight (67%) patients underwent prior liver resection. One (8%) patient received prior transarterial chemoembolization.

### Radiation treatment volumes

**Figure 2.**
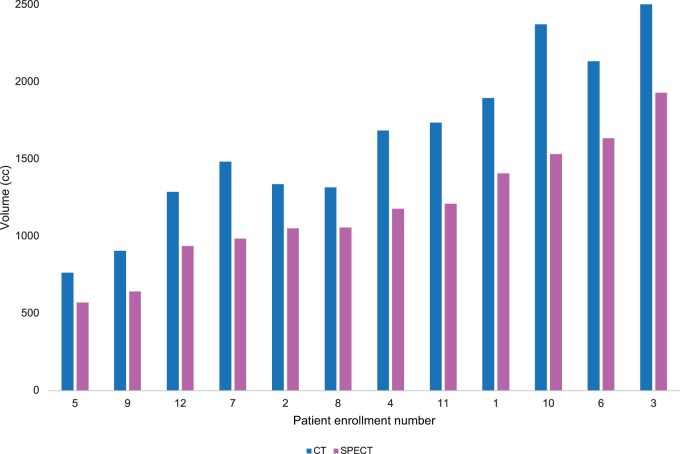
Computed tomography (liver gross tumor volume) vs single-photon emission computed tomography liver volume. CT = computed tomography; SPECT = single-photon emission computed tomography.

**Figure 3.**
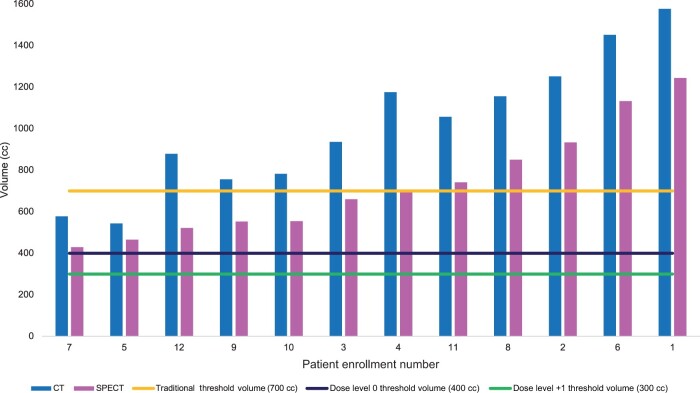
Liver volume spared below threshold dose constraint. See Table 1 for the dose constraint levels. CT = computed tomography; SPECT = single-photon emission computed tomography.

As shown in Table 3, of the patients, 9 (75%) were treated with photon intensity modulated radiation therapy (IMRT), while 3 (25%) were treated with proton therapy at the discretion of the treating radiation oncologist. The median gross tumor volume was 36 cc (range = 2-651 cc). The median CT anatomical nontumor liver gross tumor volume was 1584 cc (range = 764-2699 cc), and the median SPECT functional liver volume was 1117 cc (range = 570-1928 cc), with a Pearson correlation coefficient of 0.98 (*P* < .001) (Figure 2). The median nontarget CT liver volume below the volumetric dose constraint was 997 cc (range = 544-1576 cc). The median nontarget SPECT functional liver volume below the volumetric dose constraint was 684 cc (range = 429-1244 cc) (Figure 3). The mean dose to nontarget liver ranged from 7.2 to 28.8 Gy (median = 16.3 Gy). The mean dose to nontarget functional liver ranged from 7.5 to 23.9 Gy (median = 15.5 Gy). The median volume of the gross tumor volume receiving 95% of the prescribed dose was 99.85% (range = 80%-100%). The number of lesions treated ranged from 1 to 7 (Table 3).

**Table 3.** Tumor, liver, and radiation treatment volumes^a^

Patient enrollment number	Treatment modality	Dose, Gy or Gy(RBE)	Gross tumor volume, cc	Liver-gross tumor volume per CT, cc	Functional liver volume per SPECT, cc	Nontarget liver volume below threshold dose constraint per CT, cc	Nontarget liver volume below threshold dose constraint per SPECT, cc	Mean dose to nontarget liver, Gy or Gy(RBE)	Mean dose to nontarget functional liver, Gy or Gy(RBE)	Gross tumor volume V95, %	Number of lesions treated
1	IMRT	100	20	1895	1407	1576	1244	15.4	13.7	97.3	1
2	IMRT	75	2	1337	1051	1251	934	7.2	7.5	100.0	1
3	IMRT	100	163	2699	1928	936	660	20.5	17.3	100.0	6
4	IMRT	67.5	33	1685	1177	1175	707	19.7	21.5	100.0	1
5	IMRT	75	13	764	570	544	466	13.9	13.1	100.0	2
6	IMRT	67.5	51	2132	1635	1451	1133	20.8	19.8	100.0	2
7	Protons	75	23	1483	984	578	429	10.7	10.4	99.7	7
8	Protons	67.5	38	1317	1056	1156	850	10.7	11.3	100.0	1
9	IMRT	67.5	44	906	641	756	552	12.8	11.7	96.2	2
10	IMRT	75	11	2373	1532	782	554	28.8	23.9	98.9	6
11	IMRT	67.5	651	1736	1210	1057	741	23.4	22.2	80.0	1
12	Protons	67.5	76	1287	937	879	522	17.1	18.8	99.6	3

a CT = computed tomography; IMRT = intensity modulated radiation therapy; RBE = relative biological effectiveness; SPECT = single-photon emission computed tomography; V95 = percent volume that received at least 95% of the prescription dose.

### Dose-limiting toxicities

None of the 3 patients treated in dose level 0 and none of the 9 patients treated in dose level +1 (see Table 1) experienced any dose-limiting toxicities at 6-8 week and 6 month follow-up. The median peak total bilirubin after radiotherapy was 0.9 mg/dL (range = 0.5-1.7 mg/dL) vs baseline median 0.7 mg/dL (range = 0.4-1.9 mg/dL).

### Disease response and survival

At the time of initial restaging, imaging showed a response in the treated liver disease in 9 patients, stable liver disease in 1 patient, out-of-field liver progression in 4 patients, and distant progression in 5 patients.

Median follow-up was 20 months (range = 8-60 months). Eight patients ultimately developed an in-field recurrence, 5 patients developed an out-of-field liver recurrence, and 9 patients developed a distant recurrence. Distant recurrences occurred in the lungs (n = 7), lymph nodes (n = 3), peritoneum (n = 3), and adrenal gland (n = 1). Eight patients have died as of December 15, 2023.

**Figure 4.**
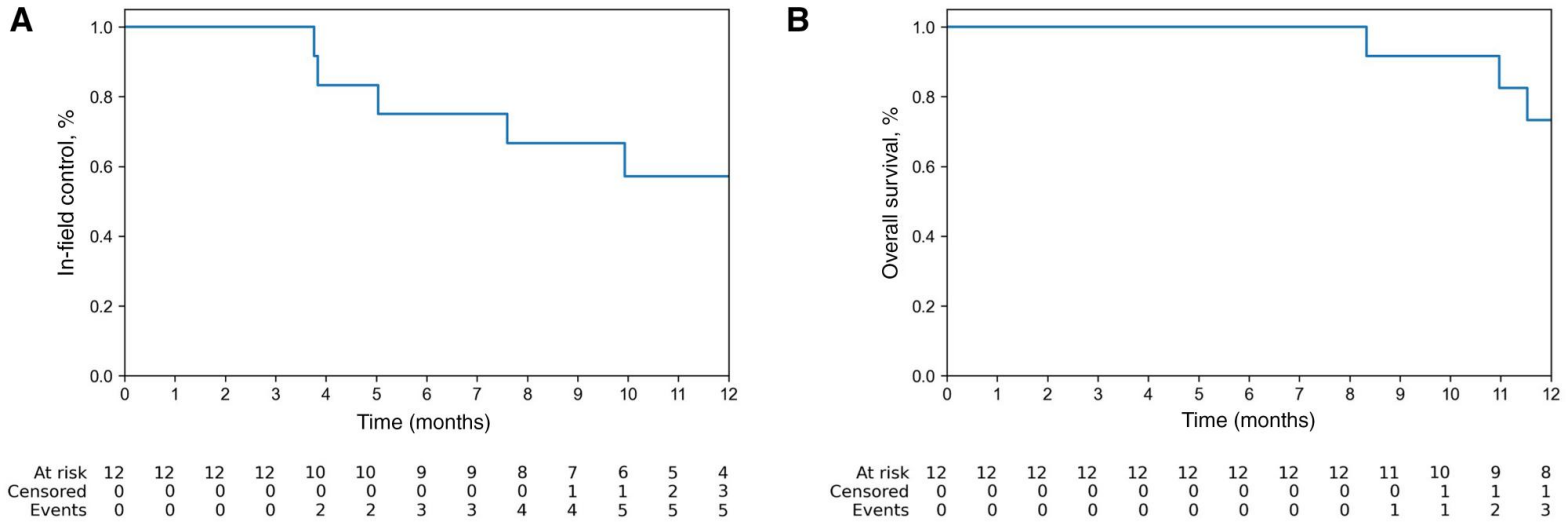
Kaplan–Meier curves for in-field control (**A)** and survival (**B**).

The 1-year in-field control rate was 57% (95% confidence interval [CI] = 35% to 94%), and 1-year overall survival was 73% (95% CI = 52% to 100%) (Figure 4).

## Discussion

In this prospective phase I study, liver-directed radiotherapy was safely delivered to high doses when incorporating functional SPECT into the radiation treatment planning process for patients with preserved liver function but low functional liver volume after receiving prior hepatotoxic chemotherapy or undergoing prior liver resection. There were no dose-limiting toxicities. Furthermore, even when using more aggressive radiation dose constraints compared with traditional liver constraints, no patients experienced radiation induced liver disease.

Although liver volume per SPECT was strongly correlated with liver volume per CT in our cohort (Pearson correlation coefficient of 0.98; *P* < .001), the additional benefit of SPECT is that it provides a spatial mapping of functional liver that can be directly incorporated into personalized radiation treatment planning and preferentially spare healthy tissue.

Specifically, in our clinical practice, we have found that SPECT provides further reassurance in patients with smaller liver volumes in whom we are unable to spare at least 700 cc of anatomic liver volumes per CT from a hepatotoxic dose of radiotherapy. Conventionally, these patients would not be candidates for liver-directed radiotherapy.

Up to 70% of total liver volume (standardized to body surface area) in patients previously treated with chemotherapy can be safely resected without inducing liver failure (18). Although it has been estimated that 500 cc liver may be spared (assuming 25% of an average volume of 2000 cc), Rusthoven et al. (1) used a more conservative constraint sparing 700 cc of normal liver from a hepatotoxic dose in a phase I-II trial of stereotactic body radiotherapy for liver metastases.

However, our experience suggests there is potential ability to be more aggressive with our constraints when treating with liver-directed radiotherapy. Two patients in this study were spared hepatotoxic doses to 544 cc and 578 cc of anatomic liver per CT and 466 cc and 429 cc of functional liver per SPECT (Figure 1) and did not experience any dose-limiting toxicities or develop radiation-induced liver disease. The use of SPECT guidance in these cases where the spared CT-based anatomic liver volume is below 700 cc provides additional reassurance that may allow expansion of eligibility for liver-directed radiotherapy. Notably, in this study, 5 patients received high-dose radiation to treat liver metastases and large volumes of nontumor liver because they were unable to undergo 2-stage hepatectomy (19) (examples in Figure 1). To our knowledge, this is the first prospective demonstration of the safety of this approach using SPECT imaging for radiation treatment planning. Furthermore, this data support the idea that the liver is a parallel structure. Based on our results and on prior studies (15), this suggests that sparing a critical volume of nontumor liver from injury is the primary determinant of liver toxicity. This finding may have broader applicability for other liver-directed therapies, including Yttrium-90, hepatic arterial infusion, and ablation techniques.

Moreover, SPECT would provide additional value if there were a large discrepancy between anatomic CT-based and functional SPECT-based liver volumes so that it would appear it was safe to treat based on CT alone, but in reality, the functional liver volume was much lower. Further refinement of the definition of functional liver using SPECT is needed. The 40% maximum intensity threshold per SPECT was selected based on prior studies published at the time of protocol development. However, more recent studies have developed thresholds optimized for Child–Pugh classification and association with clinical measures of liver function (12). This trial serves as a basis for further study to more robustly evaluate the value of SPECT. One approach would be to stratify patients into risk groups for radiation-induced liver failure and incorporate SPECT into the radiation treatment planning process for high-risk patients.

A limitation of this study is the question of generalizability of the results to other patient populations. The current study was homogenous in terms of histology as all patients had colorectal liver metastases, though this is a common indication for the study’s inclusion criteria of receiving hepatotoxic chemotherapy. Among patients with colorectal liver metastases who have been treated with ablative radiotherapy, the 1-year local control ranges from 50% to 95%, with a pooled estimate of 67% (1,20-24). By comparison, the 1-year local control in our study was 57%. This may potentially be attributed to the high proportion (83%) of patients with TP53 mutations. Of these patients, 3 had both TP53 and KRAS mutations, which is associated with worse oncologic outcomes (25,26). In one prospective study investigating the role of proton-based ablative radiotherapy for liver metastases, the 1-year local control was inferior in patients with *KRAS* mutations vs those with *KRAS* wild-type tumors (43% vs 72%, respectively), in patients with *TP53* mutations vs those without (46% vs 71%, respectively), and in patients with both *KRAS* and *TP53* mutations compared with those without (20% vs 69%, respectively) (27). Similar findings were observed in another cohort of patients with metastatic colorectal cancer who received liver-directed ablative radiotherapy (28). Notably, target coverage in our study was excellent while allowing for safe liver sparing, which suggests that reduced local control may have been more a product of biology rather than radiation technique.

As colorectal liver metastases may be more radioresistant, a multi-institutional analysis suggests that a higher radiation dose is needed to achieve local control (21). To treat these lesions to a higher dose, a higher volume of liver may potentially receive a hepatotoxic dose. The addition of SPECT may serve to optimize normal liver sparing in these cases. Although the dose and fractionation employed may be more unique to our institution, stereotactic body radiotherapy is now a more commonly used technique for treating liver oligometastases (29). A similar treatment planning approach incorporating the functional liver volume per SPECT could be employed in this setting.

Furthermore, the study design did not specify an upper limit on liver volume as an exclusion criterion, so only 2 patients had a nontarget liver volume below threshold dose constraint per CT below 700 cc. Further study is needed to assess generalizability to patients with severely limited residual liver volume.

Additional caveats to acknowledge include the limited number of patients in this cohort, challenges of registering the functional liver volume per SPECT obtained during free breathing to the breath hold treatment planning CT, and subsequent challenge with calculating the actual accumulated dose to the functional liver, heterogeneity in radiation treatment modality and only 2 patients having tumors larger than 100 cc.

Our prospective data suggest that liver-directed radiotherapy can be safely delivered to high doses when incorporating functional SPECT image guidance into the radiation treatment planning process, which may enable sparing of lower volumes of liver than traditionally accepted in patients with preserved liver function. Additional studies are needed for further validation.

## Data Availability

The data underlying this article are available in the article.
